# BLAB2CancerKD: a knowledge graph database focusing on the association between lactic acid bacteria and cancer, but beyond

**DOI:** 10.1093/database/baad036

**Published:** 2023-05-23

**Authors:** Yi Jing, Baiyang Feng, Jing Gao, Jin Li, Ganghui Zhou, Zhihong Sun, Yufei Wang

**Affiliations:** Faculty of Science, The University of New South Wales, High Street, Sydney, New South Wales 2052, Australia; Inner Mongolia Autonomous Region Key Laboratory of Big Data Research and Application for Agriculture and Animal Husbandry, Zhaowuda Road No. 306, Hohhot 010018, China; Inner Mongolia Autonomous Region Key Laboratory of Big Data Research and Application for Agriculture and Animal Husbandry, Zhaowuda Road No. 306, Hohhot 010018, China; College of Computer and Information Engineering, Inner Mongolia Agricultural University, Erdos East Street No. 29, Hohhot 010011, China; Inner Mongolia Autonomous Region Key Laboratory of Big Data Research and Application for Agriculture and Animal Husbandry, Zhaowuda Road No. 306, Hohhot 010018, China; College of Computer and Information Engineering, Inner Mongolia Agricultural University, Erdos East Street No. 29, Hohhot 010011, China; Inner Mongolia Autonomous Region Big Data Center, Chilechuan Street No. 1, Hohhot 010091, China; Inner Mongolia Autonomous Region Key Laboratory of Big Data Research and Application for Agriculture and Animal Husbandry, Zhaowuda Road No. 306, Hohhot 010018, China; College of Computer and Information Engineering, Inner Mongolia Agricultural University, Erdos East Street No. 29, Hohhot 010011, China; Inner Mongolia Autonomous Region Key Laboratory of Big Data Research and Application for Agriculture and Animal Husbandry, Zhaowuda Road No. 306, Hohhot 010018, China; College of Computer and Information Engineering, Inner Mongolia Agricultural University, Erdos East Street No. 29, Hohhot 010011, China; College of Food Science and Engineering, Inner Mongolia Agricultural University, Zhaowuda Road No. 306, Hohhot 010018, China; The Affiliated Hospital of Inner Mongolia Medical University, Tongdao North road No.1, Hohhot 010050, China

## Abstract

In a broad sense, lactic acid bacteria (LAB) is a general term for Gram-positive bacteria that can produce lactic acid by utilizing fermentable carbohydrates. It is widely used in essential fields such as industry, agriculture, animal husbandry and medicine. At the same time, LAB are closely related to human health. They can regulate human intestinal flora and improve gastrointestinal function and body immunity. Cancer, a disease in which some cells grow out of control and spread to other body parts, is one of the leading causes of human death worldwide. In recent years, the potential of LAB in cancer treatment has attracted attention. Mining knowledge from the scientific literature significantly accelerates its application in cancer treatment. Using 7794 literature studies of LAB cancer as source data, we have processed 16 543 biomedical concepts and 23 091 associations by using automatic text mining tools combined with manual curation of domain experts. An ontology containing 31 434 pieces of structured data is constructed. Finally, based on ontology, a knowledge graph (KG) database, which is called Beyond ‘Lactic acid bacteria to Cancer Knowledge graph Database’ (BLAB2CancerKD), is constructed by using KG and web technology. BLAB2CancerKD presents all the relevant knowledge intuitively and clearly in various data presentation forms, and the interactive system function also makes it more efficient. BLAB2CancerKD will be continuously updated to advance the research and application of LAB in cancer therapy. Researchers can visit BLAB2CancerKD at.

**Database URL** http://110.40.139.2:18095/

## Introduction

Lactic acid bacteria (LAB) is a generic term for Gram-positive bacteria that produce lactic acid, which contain over 60 genera (1, 2). LAB are closely related to human life and are widely used in food processing, farming, animal husbandry and medicine production (3). At the same time, LAB have numerous beneficial effects on the human body, including regulating the microbial community in the gastrointestinal tract, improving immune response, controlling endotoxin and improving the body’s immunity (4, 5). Cancer, also called malignant tumor, is a group of diseases involving abnormal cell growth that may invade or spread to other body parts (6, 7). There are more than 100 types of cancers that affect human health (8). With nearly 10 million deaths by 2020, cancer remains one of the major challenges of the 21st century (9). Recent studies have shown that LAB can inhibit the onset or progression of cancer in various ways, which has potential clinical value in cancer prevention and treatment (10).

There are many existing databases that are related to LAB. Random amplified polymorphic DNA polymerase chain reaction (RAPD-PCR) fingerprint databases (11) have identified and typed about a thousand LAB isolated from cheese by species-specific PCR and RAPD-PCR and constructed a LAB fingerprint database. It can identify newly isolated strains, improve previous identification or identify LAB strains isolated from other food ecosystems. LABiocin database (12) is a database especially designed for LAB’s bacteriocin, including name, category, amino acid, nucleic acid sequence of bacteriocin, target microorganism, source, state of production strain and its culture conditions, as well as extraction and purification methods. Similarly, A web-based bacteriocin genome mining tool (BAGEL) (13) also contains information about LAB’s bacteriocin. GelCompar II database (14) contains DNA fingerprints of EcoRI fragments stained with ethidium bromide of total LAB DNA isolated by routine agarose gel electrophoresis. LAB-Secretome (15) stores, visualizes and updates extracellular and surface-associated proteins and Lacto bacillales Cluster of Ortholog Groups (LaCOG) of LAB. Currently, there are many public cancer databases whose primary purpose is to describe cancer and its characteristics, such as National Cancer Institute Thesaurus (16), one of the most commonly used cancer research ontologies, which compiles terms across all aspects of cancer research and health care. Manually handled by experts, Catalogue of Somatic Mutations in Cancer (17) is the world’s largest and most comprehensive resource for studying the effects of variations in human cancer cells. SynLethDB (18) is a comprehensive knowledge base of synthetic lethality (SL), containing an extensive collection of SL gene pairs from various sources to discover selective and sensitive anticancer drug targets. However, till now, no knowledge graph (KG) database has focused on the association between LAB and cancer.

Using LAB cancer–related scientific literature as data sources, we constructed Beyond ‘Lactic acid bacteria to Cancer’ Knowledge-graph Database (BLAB2CancerKD). This KG database focuses on, but beyond, the association between LAB and cancer. Although manual sorting of unstructured data in biomedical literature maintains a high accuracy and recall rate, this method needs to be improved in time, cost and efficiency (19). Although the existing biomedical text mining (TM) (20) tools can solve the problem of time, cost and low efficiency, the accuracy is far less than the manual way (21). Therefore, we use TM tools and web crawler technology (22) combined with a semi-automatic method of manual curation by domain experts to extract relevant structured knowledge from scientific literature, which saves time and cost and dramatically improves accuracy and recall rate. The ontology of ‘beyond lactic acid bacteria cancer’ was established, and finally, BLAB2CancerKD was constructed based on data combined with KG and web technology. The visual form and interactive user interface of the KG can help researchers quickly acquire knowledge of the association between LAB and cancer and assist researchers in finding new strategies, methods and applications of LAB in cancer treatment and prevention.

## Method

**Figure 1.**
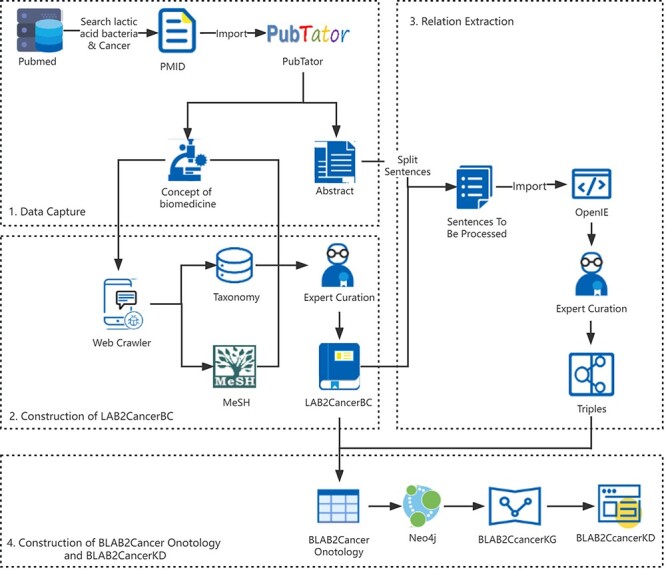
BLAB2CancerKD flow chart.

This paper used TM tools combined with more in-depth manual curation of domain experts to obtain the structured knowledge of LAB and cancer from the scientific literature. Based on these data, KGs and web technology were adopted to construct BLAB2CancerKD. The process is shown in Figure 1.

### Data source

We obtained the PubMed Unique Identifier (PMID) of 7794 related articles from PubMed (https://pubmed.ncbi.nlm.nih.gov/) by using ‘lactic acid bacteria&cancer’ as keyword. PubTator (23) is an automatic, web-based biomedical concept annotation system that provides an Application Programming Interface (API) interface to easily access and extract biomedical knowledge with a large amount of data. Biomedical entities that can be annotated contain gene/protein, disease, chemical, mutation, species and cell line. We used PubTator to obtain the relevant literature abstracts and the corresponding six biomedical concepts.

### Construction of LAB cancer–related biomedical concepts

Medical Subject Headings (MeSH) (24) is an The United States National Library of Medicine (NLM)-controlled vocabulary thesaurus used to index PubMed articles and includes 19 categories of biomedical concepts. Taxonomy database (25) has the classification and nomenclature of all organisms in public sequence databases. Based on MeSH and taxonomy, we further supplemented and improved the biomedical concepts given by PubTator by using crawler technology combined with in-depth expert curation and obtained an entity dictionary of biomedical concepts related to LAB and cancer, which is called LAB cancer–related biomedical concepts (LAB2CancerBC).

We tend to focus on the knowledge of LAB cancer and its related diseases; hence, the division of diseases into specific strains is more detailed. We subdivided the disease category according to the ‘Diseases Category’ in MeSH. Regarding the categories of specific strain entities, we marked the strains at the ‘genus’ level according to the classification standard of taxonomy database to more clearly show the association between different strains and cancer and its related diseases.

### Relation extraction

We use LAB2CancerBC as a reference and utilize Python regular expressions (https://docs.python.org/3/library/re.html) to split the relevant abstracts into pre-processing statements. Finally, we use the Open Domain Information Extraction (26) (OpenIE) System and expert curation to complete the relation extraction (RE) task. OpenIE is a RE tool that can extract relational tuples from text without a given training corpus. For example, the sentence ‘I like playing basketball’ can be extracted to obtain a triple: (I, like playing, basketball). We prefer this kind of relationship extraction method that keeps the original semantic structure of scientific literature the same rather than defining the type of relationship in advance to supporting deeper curation and obtaining structured data with more prosperous association relations.

### Manual curation of experts

**Figure 2.**
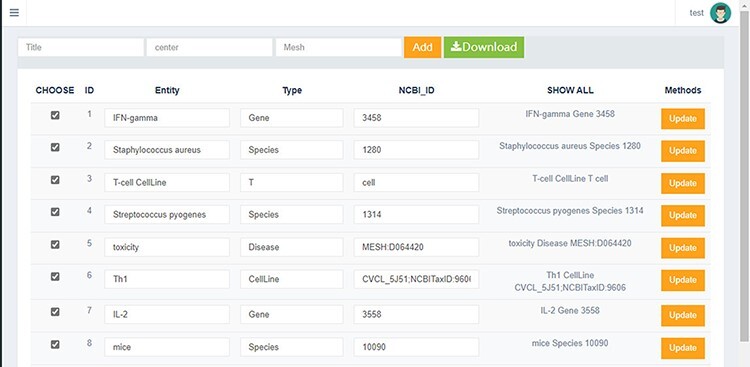
System of manual curation of experts.

We have implemented a system for the manual curation of experts, which visualizes all structured data and frees experts from tedious file operations. The system is shown in Figure 2. In the construction task of LAB2CancerBC, the domain experts mainly carry out entity curation work on four aspects, which are unlabeled concepts, incorrectly labeled concepts, incompletely labeled concepts and specific strains that were unsuitably processed by PubTator. At the same time, the concept entity from the manual curation of experts will be continuously updated in LAB2CancerBC to reduce labor costs continuously.

#### Unlabeled concepts

Barrett’s esophagus is a marker for severe reflux and precursor to adenocarcinoma of the esophagus. It is also available in MeSH. We labeled it as ‘Barrett’s esophagus, neoplasms, MESH: D001471’ and ‘Barrett’s esophagus, digestive system disease, MESH: D001471’. Meanwhile, we also focus on biomedical concepts such as analytical, diagnostic and therapeutic techniques and equipment category, phenomena and processes category and technology and food and beverages category, unlabeled by PubTator. These biomedical concepts need to be processed by experts as well.

#### Incorrectly labeled concepts

LF-HFY06 is the abbreviation of *Limosilactobacillus fermentum* HFY06. PubTator annotated it as a cell line concept, which we corrected to ‘LF-HFY06, *Limosilactobacillus*, 1613’. Lipopolysaccharide (LPS) is a component of the outer wall of the cell wall of Gram-negative bacteria, and it is also a kind of endotoxin. However, PubTator identified it as a disease called lip-pits syndrome or the house mouse Tlr4 toll–like receptor 4 gene. We corrected it to ‘LPS, Chemical, MESH: D008070’.

#### Incompletely labeled concepts

PubTator only labels ‘cancer’ for oral cancer, which is not comprehensive enough for our work. Hence, we label it ‘oral cancer, neoplasms, MESH: D009062’ and ‘oral cancer, stomatognathic disease, MESH: D009062’.

#### Annotation of the genus of specific LAB strains

We labeled *Lactobacillus fermentum* HFY06 as ‘*Lactobacillus fermentum* HFY06, *Limosilactobacillus*, 1613’. We labeled *Lactobacillus rhamnosus* ATCC 7469 as ‘*Lactobacillus rhamnosus* ATCC 7469, *Lacticaseibacillus*, 47715’.

In the RE task, the structured triples extracted by OpenIE also have some inaccuracies, such as redundancy and missing. In order to ensure the accuracy of RE results, we implement further manual curation based on OpenIE extraction results to improve the accuracy of data. For instance, the sentence ‘22611376|t|Cholesterol-lowering probiotics as potential biotherapeutics for metabolic diseases.’ has no extracted result. Hence, we extracted the triples manually and obtained ‘cholesterol-lowering probiotics, as potential biotherapeutics for metabolic diseases’.

### Construction of BLAB2Cancer ontology and BLAB2CancerKG

We combined LAB2CancerBC with triples to obtain a structured BLAB2Cancer ontology containing relevant biomedical concepts, categories, National Center for Biotechnology Information (NCBI)_ids, associations and corresponding statements. Neo4j (https://neo4j.com/) is a graph database management system with local graph storage and processing functions. Data are stored in the form of nodes, edges or attributes. We use Python scripts to import BLAB2Cancer ontology into Neo4j to obtain LAB cancer KG, BLAB2CancerKG.

### Construction of BLAB2CancerKD

We developed BLAB2CancerKD based on BLAB2CancerKG. The front end of BLAB2CancerKD is constructed by using Python Django web frame (https://www.djangoproject.com/), and the back end is implemented by using Neo4j and MySQL (https://www.mysql.com/cn/). We use pyecharts (https://pyecharts.org/#/) and cytoscape.js (https://js.cytoscape.org/) to visualize the data of KG.

## Result

### LAB2CancerBC

**Figure 3.**
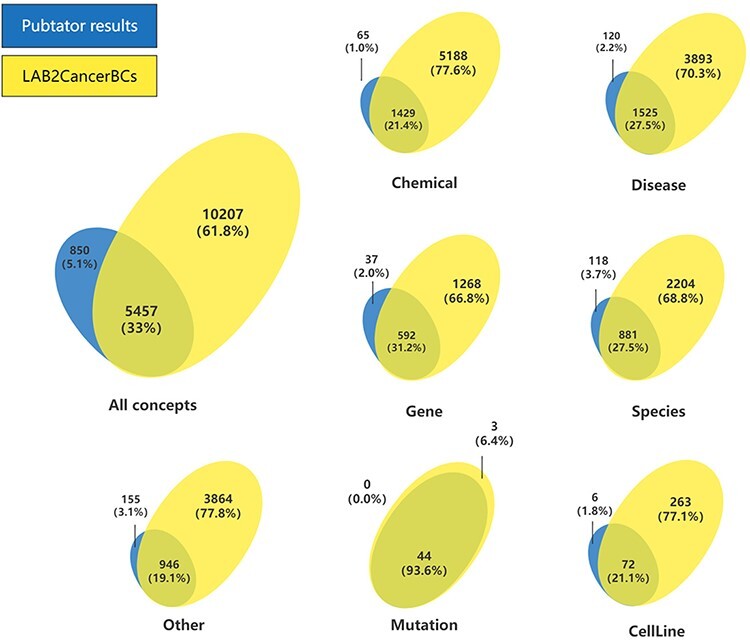
Comparison of annotation of PubTator with annotation of LAB2CancerBC.

**Figure 4.**
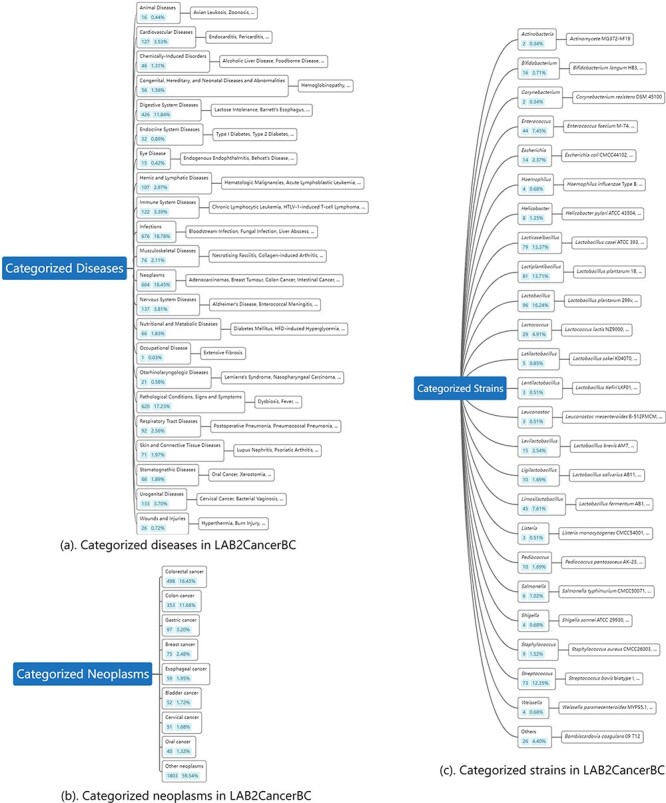
Statistics of major entity categories in LAB2CancerBC.

A combination of PubTator, web crawler techniques and hand curation by domain experts resulted in LAB2CancerBC with 157 species of 19 020 relevant biomedical concepts. The partial content of LAB2CancerBC is shown in Table 1. Figure 3 shows the comparison of the annotation of PubTator with the annotation of LAB2CancerBC. It can be clearly seen from the figure that LAB2CancerBC after processing is more abundant in the number of entities and categories. Figure 4a shows the tree diagram of categorized diseases. The category ‘neoplasms’ accounted for the largest proportion, up to 18.48%, which involves colon cancer, Barrett’s esophagus, breast tumor, gastric cancer, etc., then come pathological conditions, signs and symptoms, including various types of bacteremia caused by various types of reasons, sepsis, diarrhea, etc. Figure 4b shows the tree diagram of categorized neoplasms since we mainly focus on cancer. Figure 4b shows that the percentage of colorectal cancer (CRC) is the biggest, 16.45%. Then, colon cancer accounts for 11.66%, gastric cancer accounts for 3.20%, etc. We merged the cases that have small quantities into ‘other neoplasms’. Figure 4c shows the tree diagram of categorized strains. *Lactobacillus* accounted for the most significant proportion, including *Lactobacillus plantarum, Lactobacillus acidophilus*, *L. fermentum*, etc., then comes *Lacticaseibacillus*, mainly including *Lactobacillus casei, Lactobacillus paracasei*, *L. rhamnosus*, etc.

**Table 1.** Partial content of LAB2CancerBC

Entity name	Entity type	NCBI_ID
Lymphangioleiomyomatosis	Immune system diseases	MESH: D018192
enteritis	Digestive system disease	MESH: D004751
*L. pentosus* strains LB2F2	*Lactiplantibacillus*	1589
*L. rhamnosus* P1	*Lacticaseibacillus*	47 715
Bacteremia	Infections	MESH: D016470

### LAB2Cancer ontology and BLAB2CancerKG

**Figure 5.**
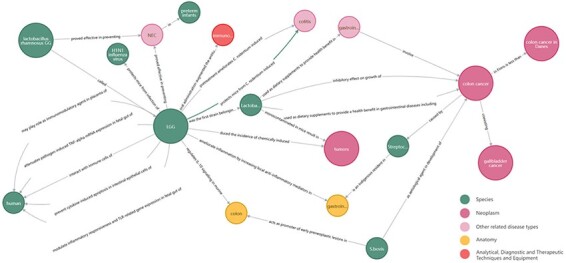
Part of BLAB2CancerKG instance.

Combined with OpenIE for relationship extraction and expert curation, 31 434 structural triples were obtained. We combined LAB2CancerBC with triples to obtain LAB2Cancer ontology. Table 2 shows partial results of LAB2Cancer ontology. Entity 1 of each dataset in the structured triples is Node 1, the association relationship is the edge, and Entity 2 is Node 2. Each node is attached with the entity category and NCBI_ID in LAB2CancerBC. BLAB2CancerKG is constructed based on Neo4j, which contains 16 543 nodes, 23 091 edges and 72 720 attributes. A partial example of ‘LGG’, that is, ‘*L. rhamnosus* GG’, as the central node, is shown in Figure 5.

**Table 2.** Partial results of LAB2Cancer ontology

Entity 1	Entity 1 classification	Entity 1 ID	Relation	Entity 2	Entity 2 classification	Entity 2 ID	Sentence
*L. helveticus* R389	*Lactobacillus*	1587	Decreasing Interleukin-6 in	Mammary glands	Anatomy	MESH:D042361	20 550 747|milk fermented with…
Hepatic abscess	Digestive system disease	MESH:D008100	As first manifestation of	Renal carcinoma	Neoplasms	MESH:D002292	12 073 672|t|[Hepatic abscess…
*L. lactis* 332	*Lactococcus*	1358	Caused an accumulation of	Macrophages	Anatomy	MESH:D008264	9 564 792|Intraperitoneal injection of…
Invasive Pneumococcal Disease (IPD)	Immune system disease	MESH:C564468	Increase in population with	Multiple myeloma	Cardiovascular disease	MESH:D009101	20 429 967|Compared to…
CRIP1	Gene	1396	Activated by	*E. faecalis*	Species	1351	27 836 662|t|CRIP1, a novel…

### BLAB2CancerKD

**Figure 6.**
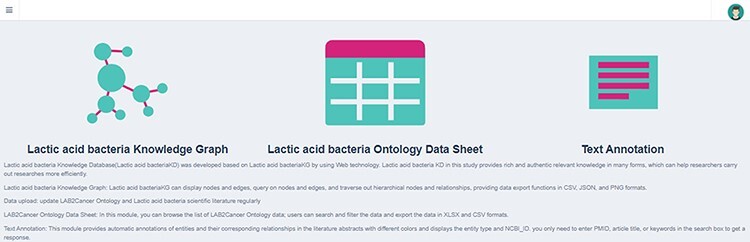
Home page of BLAB2CancerKD.

**Figure 7.**
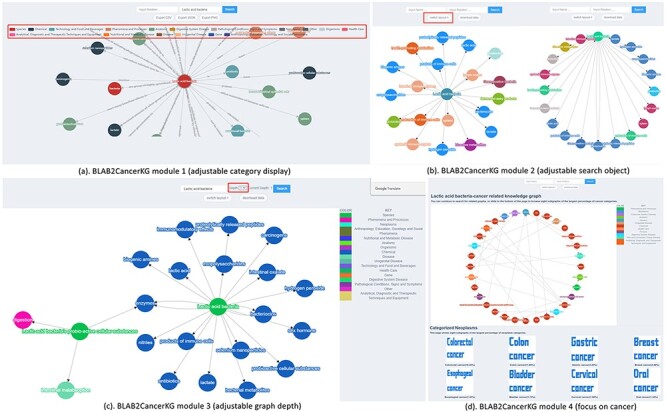
BLAB2CancerKG module.

**Figure 8.**
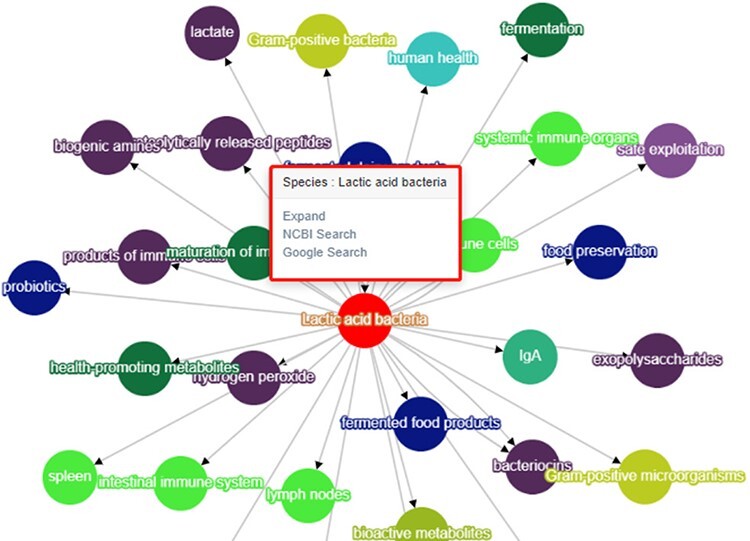
Node expansion of the KG module.

**Figure 9.**
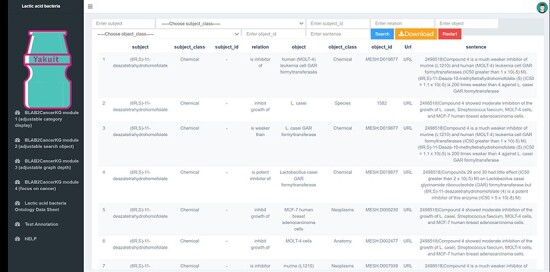
BLAB2Cancer sheet module.

**Figure 10.**
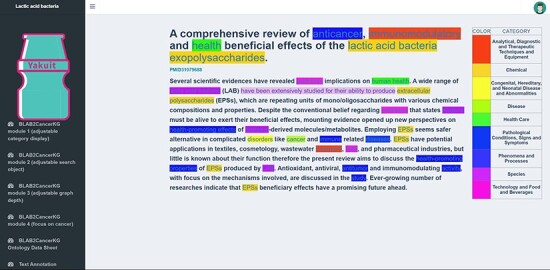
BLAB2CancerKD overview.

BLAB2CancerKD can quickly browse and inquire about LAB cancer knowledge and download data. It includes a KG module, a data list module and a text annotation module. Users can access BLAB2CancerKD at http://110.40.139.2:18095/. Its home page is shown in Figure 6, which is mainly divided into the following three functional modules:

- BLAB2CancerKG module: This module speeds up the interaction between users and data and can fully display biomedical concepts, entity categories, NCBI_ID and the association between concepts, while providing search and data export functions. We have divided four submodules according to specific functions.

- BLAB2CancerKG module 1 (adjustable category display): This module mainly uses data display as the main function. Users can search for ‘concept’ or ‘association’ to get KG and then click the entity label legend to hide or display other entities of different categories. The module supports data export in three forms: .csv, .JavaScript Object Notation (.JSON) and .Portable Network Graphics (.PNG), as shown in Figure 7a.
- BLAB2CancerKG module 2 (adjustable search object), as shown in Figure 7b. After the user obtains the submap through searching, the submodule can switch between two different KG display forms by clicking the ‘switch layout’ button and support data download in .Comma-Separated Values (.CSV) format. In addition, researchers can click on a node to view the KG with this node as the center node and provide Google search and NCBI search at the same time, as shown in Figure 8.
- BLAB2CancerKG module 3 (adjustable graph depth) allows users to view subgraphs of different node depths. We set it in the range of 1–4 layers. Other functions of this module are consistent with Module 2, as shown in Figure 7c.

(4) BLAB2CancerKG module 4 (focus on cancer), in order to focus more on cancer, in this module, we have limited the scope of BLAB2CancerKG, that is, to ensure that all the information users search for under this module is related to cancer, so that users can have more efficient access to cancer-related information. The page also provides a submap of the eight tumor categories that account for the largest proportion, as shown in Figure 7d.

- BLAB2Cancer Sheet module: This module displays all BLAB2Cancer ontology structured data in a list form, provides search and data export functions and provides PubMed links for traceability verification. The module is shown in Figure 9.
- Text annotation module: This module includes all 7794 scientific literature abstracts related to LAB and cancer. Users can search corresponding literature abstracts through PMID or keywords. This module highlights all relevant biomedical concepts, and the corresponding category and NCBI_ID can be seen when the cursor is placed on the corresponding entity concepts. The module is shown in Figure 10.

## Discussion

**Figure 11.**
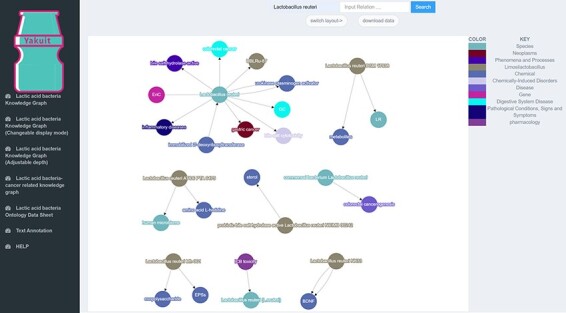
KG of *L. reuteri*.

Accessing LAB and cancer-related literature through PubMed retrieval requires researchers to spend a lot of learning costs to obtain the relevant knowledge, which is time-consuming and laborious. Our BLAB2CancerKD integrates unstructured knowledge in the literature in multiple formats, allowing researchers to access the knowledge more easily and quickly. For example, suppose researchers would like to get a quick overview of *Lactobacillus reuteri*, they can search for *L. reuteri* in the Lactic Acid Bacteria Knowledge Graph interface, and KD will show all the specific strains affiliated with this species in the graph related to other entities, as shown in Figure 11.

We will describe in detail how BLAB2CancerKD accelerates knowledge acquisition and promotes knowledge discovery in ‘*Lactobacillus* for cancer treatment or prevention’ and explain why we say ‘beyond’ based on overall data and specific examples.

### LAB and cancer therapy

BLAB2CancerKD contains 50 genera, including the core *Lactobacillus, Connostrea*, and *Amphicoccus*, as well as the more peripheral *Aerococcus, Botulus, Enterococcus*, etc. As experts pay more attention to the potential application of specific strains in cancer treatment, we collected 591 specific strains. The association between these LAB and cancer and related diseases can be clearly seen through the KG and ontology data list. For example, we can see that *L. rhamnosus* GG (LGG) can reduce tumor load in a Cluster of Differentiation 8 T-cell-dependent manner (27). *Lactobacillus* E6-1 inhibited Cal-27 and induced apoptosis of oral cancer cells (28). *L. rhamnosus* Probio-M9 can treat inflammation and colitis-associated tumorigenesis by regulating intestinal environment (29). Researchers can quickly learn from BLAB2CancerKD’s multiple data presentation methods, in which LAB strains have potential in cancer therapy and adjuvant cancer therapy, and then carry out targeted research work without the need to read an enormous amount of scientific literature in advance, thus improving the efficiency of scientific research.

### LAB and cancer prevention

LAB, including specific strains, can help prevent cancer. We have also collected relevant data. For example, eating *Bifidobacterium Longum* SPM1207 can help to improve mild to moderate hypercholesterolemia to prevent CRC (30). *Lactobacillus salivarius* Ren isolated from centenarians living in Bma, China (the longevity land of the world), can prevent dimethylhydrazine-induced CRC by protein kinase B inhibition (31). *Lactobacillus reuteri* ATCC-PTA-6475 can inhibit mammary carcinogenesis (32). *Lactobacillus casei* BL23 can prevent colitis-associated CRC (33). *Lactobacillus casei* strain Shirota could inhibit 3-methylcholanthrene-induced tumorigenesis (34). Prophylactic administration of *L. casei* ATCC 393 delays the onset of cancer (35).

### Microbial communities and cancer

Through BLAB2CancerKG, we learned that microbial communities might also determine human health and cancer treatment. For example, compared with the gut microbiota of healthy people, disturbed microbial balance is often observed in patients with CRC, who have more *Enterococcus faecalis*, but less *Lactobaccilus acidophilus* and *Lactoacillus palntarom* (36, 37). A profusion of *Fusobacterium nucleatum* CTI-2 and a decrease in *Streptococcus pneumoniae* were found in oral cancer patients’ oral microbiota. This knowledge emphasizes that modulation of microbiota balance can reduce cancer incidence and targeting microbiome analysis can potentially complement existing cancer screening approaches.

### LAB and cancer-related diseases

LAB can better cope with cancer complications, reduce the pain of cancer patients and improve the quality of life of patients. BLAB2CancerKD lists 1919 cancer-related diseases and summarizes how LAB respond to these diseases. For example, oral mucositis (OM) is a common complication in head and neck cancer chemotherapy. *Lactobacillus reuteri* DSM 17 938 and *L. reuteri* ATCC PTA 5289 can improve OM (38). *Lactobacillus casei* YIT9018 can prevent leukopenia (39) during radiotherapy in cancer patients, is an adjuvant immunotherapy and can be used in combination with radiotherapy.

Not all LAB are good for human health. For example, *Streptococcus pyogenes*, or Group A *Streptococcus*, can cause acute pharyngitis (40) and necrotizing fasciitis (41). *Enterococcus faecalis* infection may be involved in the progression of chronic pancreatitis and eventually lead to the development of pancreatic cancer (42).

### Why ‘Beyond’? Other related entities

We describe BLAB2CancerKD as ‘beyond’ because it also contains concepts such as gene, chemical, cell line, phenomena and process, technology and food and beverages, analytical, diagnostic and therapeutic techniques and equipment, etc. These rich entities can more comprehensively and systematically show other extended concepts and relationships of LAB or cancer and expand scientific research ideas. This structured knowledge in KG may hold promise for uncovering hidden interactions between LAB and cancers. For example, *Lactobacilli* R389 fermented milk could delay breast cancer growth by reducing serum IL-6 and increasing IL-10 in breast and tumor-infiltrating immune cells (43). Some probiotic *Lactobacillus* species in kimchi, such as *Anaerobia* and *L. plantarum*, can not only be used as antibacterial agents in foods but also develop functional foods to reduce the risk of colon cancer (44, 45). *Lactobacillus plantarum* 299 v can improve the nutritional status, enteral nutrition tolerance and quality of life in cancer patients (46). Lunasin, a cancer-preventive peptide, is synthesized by LAB during sourdough fermentation (47). Regular intake of aspirin and other Nonsteroidal Antiinflammatory Drugs reduces the risk of CRCs and adenomas (48). Catechol can inhibit breast cancer cell proliferation and mammosphere formation (49).

### Explore and discovery

While searching through PubMed, researchers need to read a large amount of literature and finally summarize and integrate all the information, which is time-consuming and laborious. Compared with searching from PubMed, the knowledge base we built integrates the unstructured knowledge scattered in the literature together in the form of graphs, and hence, researchers can search more conveniently and quickly.

**Figure 12.**
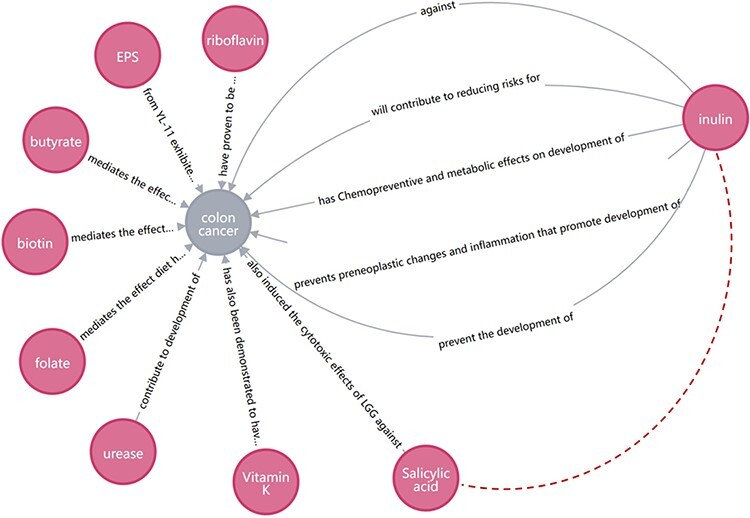
Speculation on the synergistic effect of inulin and salicylic acid.

**Figure 13.**
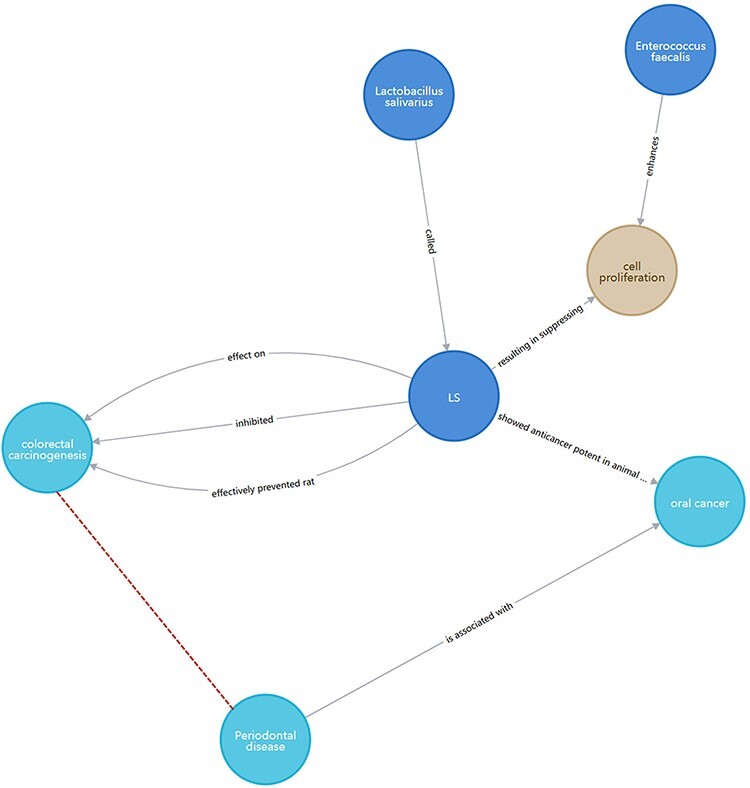
Potential association between periodontal disease and CRC.

Inference research can be carried out through KG, and potential relationships can be mined to provide ideas for researchers and give a start for subsequent scientific experiment verification. An example is given to show how BLAB2CancerKG can be used to facilitate biological discovery. Figure 12 shows that inulin can reduce risks for colon cancer, while salicylic acid can induce the cytotoxic effects of LGG against colon cancer. We speculate that inulin and salicylic acid may have a synergistic relationship. In the subsequent innovative research and development, it may be possible to consider combining inulin with salicylic acid to develop health products or drugs for specific cancers. As shown in Figure 13, *L. salivarius* inhibits colorectal carcinogenesis and shows an anticancer potential in oral cancer. Therefore, the two cancer-inducing substances may have similar properties that can be acted upon by *L. salivarius*. Meanwhile, periodontal disease is associated with oral cancer; therefore, there may be an association between periodontal disease and colorectal carcinogenesis. Patients with periodontal disease should be alerted to the occurrence of colorectal carcinogenesis along with the prevention of oral cancer.

## Conclusion

We have constructed a knowledge graph database, BLAB2CancerKD, for the lactobacillus-cancer association using text mining techniques and in-depth expert curation. As of now, it has systematically collected and displayed 31,434 structured pieces of relevant knowledge from 7,794 literature sources. All the data can be found at http://110.40.139.2:18095/, along with the Pubmed links to the corresponding literature for verification and accuracy checking.

BLAB2CancerKD provides knowledge integration for the research and application of LAB in cancer prevention and treatment. The KG, an efficient visual representation, helps to promote the scientific research process and helps researchers discover hidden and previously unknown biological associations. BLAB2CancerKD will continue to provide data support for related research, and incremental updating of data is still part of our future task. We will also combine intelligent question answering and deep learning to further explore the association between LAB and cancer and provide new biological insights.
